# Alteration of colonic excitatory tachykininergic motility and enteric inflammation following dopaminergic nigrostriatal neurodegeneration

**DOI:** 10.1186/s12974-016-0608-5

**Published:** 2016-06-13

**Authors:** Carolina Pellegrini, Matteo Fornai, Rocchina Colucci, Erika Tirotta, Fabio Blandini, Giovanna Levandis, Silvia Cerri, Cristina Segnani, Chiara Ippolito, Nunzia Bernardini, Karolina Cseri, Corrado Blandizzi, György Haskó, Luca Antonioli

**Affiliations:** Department of Clinical and Experimental Medicine, University of Pisa, Via Roma 55, 56126 Pisa, Italy; Department of Pharmaceutical and Pharmacological Sciences, University of Padova, 35131 Padova, Italy; Laboratory of Functional Neurochemistry, Center for Research in Neurodegenerative Diseases, “C. Mondino” National Neurological Institute, 27100 Pavia, Italy; Department of Medical Chemistry, Medical and Health Science Center, University of Debrecen, Debrecen, 4032 Hungary; Department of Surgery and Center for Immunity and Inflammation, Rutgers New Jersey Medical School, Newark, 07103 NJ USA

**Keywords:** 6-hydroxydopamine, Parkinson’s disease, Colonic motility, Tachykininergic neurotransmission, Substance P, Inflammation

## Abstract

**Background:**

Parkinson’s disease (PD) is frequently associated with gastrointestinal (GI) symptoms, including constipation and defecatory dysfunctions. The mechanisms underlying such disorders are still largely unknown, although the occurrence of a bowel inflammatory condition has been hypothesized. This study examined the impact of central dopaminergic degeneration, induced by intranigral injection of 6-hydroxydopamine (6-OHDA), on distal colonic excitatory tachykininergic motility in rats.

**Methods:**

Animals were euthanized 4 and 8 weeks after 6-OHDA injection. Tachykininergic contractions, elicited by electrical stimulation or exogenous substance P (SP), were recorded in vitro from longitudinal muscle colonic preparations. SP, tachykininergic NK_1_ receptor, and glial fibrillary acidic protein (GFAP) expression, as well as the density of eosinophils and mast cells in the colonic wall, were examined by immunohistochemical analysis. Malondialdehyde (MDA, colorimetric assay), TNF, and IL-1β (ELISA assay) levels were also examined. The polarization of peritoneal macrophages was evaluated by real-time PCR.

**Results:**

In colonic preparations, electrically and SP-evoked tachykininergic contractions were increased in 6-OHDA rats. Immunohistochemistry displayed an increase in SP and GFAP levels in the myenteric plexus, as well as NK_1_ receptor expression in the colonic muscle layer of 6-OHDA rats. MDA, TNF, and IL-1β levels were increased also in colonic tissues from 6-OHDA rats. In 6-OHDA rats, the number of eosinophils and mast cells was increased as compared with control animals, and peritoneal macrophages polarized towards a pro-inflammatory phenotype.

**Conclusions:**

The results indicate that the induction of central nigrostriatal dopaminergic degeneration is followed by bowel inflammation associated with increased oxidative stress, increase in pro-inflammatory cytokine levels, activation of enteric glia and inflammatory cells, and enhancement of colonic excitatory tachykininergic motility.

## Background

Gastrointestinal (GI) symptoms in Parkinson’s disease (PD), such as dysphagia, nausea, delayed gastric emptying, abdominal distension, and chronic constipation, represent prominent non-motor features of the disease, which often precede the onset of typical motor symptoms [1, 2]. Such GI disturbances contribute to the morbidity of PD, complicating its clinical management [3, 4]. Changes in enteric nervous system (ENS) have been proposed to be involved in the onset and development of digestive symptoms in PD, and there is evidence that they represent one of the earliest signs of the disease, as suggested also by the presence of α-synuclein-positive inclusions (Lewy bodies), the typical hallmarks of PD, in neurons of the myenteric plexus of PD patients [4]. However, little is known about the neurochemical, molecular and functional changes occurring in the GI tract in the presence of PD.

Both pre-clinical and human studies have shown that PD is associated with functional GI changes [2]. In particular, animals with PD-like features, induced by systemic injection of specific neurotoxins, as well as transgenic mice overexpressing mutant A53T α-synuclein, were found to display moderate GI dysfunctions, including reduced gastric emptying, prolonged GI transit time and decreased fecal output [5–7]. Other authors reported the occurrence of GI dysmotility following 6-hydroxydopamine (6-OHDA) injection into the medial forebrain bundle (MFB) of rats, a well-known procedure inducing massive degeneration of dopaminergic neurons in the substantia nigra [8]. In particular, alterations of enteric neurotransmitters (dopamine, nitric oxide, vasoactive intestinal peptide, acetylcholine), all involved in the regulation of intestinal motility, have been documented in the 6-OHDA model, thus suggesting that central dopaminergic neurodegeneration is associated with remodeling of enteric neurotransmission [9–12]. In this context, very scarce attention has been paid to the possible involvement of enteric tachykininergic pathway in the pathophysiology of gut motor dysfunctions associated with PD. This is quite surprising, in view of the evidence that intrinsic enteric tachykininergic nerves play a pivotal role in the physiological regulation of GI motility and that such system undergoes significant rearrangements under pathological conditions, including bowel motor dysfunctions associated with inflammatory disorders [13, 14].

Enteric neuronal rearrangement represents a common feature of several bowel disorders sharing the presence of an inflammatory enteric condition (e.g., inflammatory bowel diseases, diverticulitis, and irritable bowel syndrome) [15]. In this regard, it is noteworthy that processes of enteric neuroplastic remodeling [16], as well as signs of colonic tissue inflammation [17], have been observed also in PD patients, supporting the hypothesis that bowel inflammation and the related remodeling of enteric neurotransmission could contribute to the pathophysiology of intestinal motor abnormalities associated with PD. However, whether, and to what extent, inflammatory bowel alterations can occur also in experimental models of PD, and above all, whether such putative changes can stem from dopaminergic nigrostriatal degeneration, a pathological hallmark of PD, remain presently unclear.

Based on the above background, the present study was specifically designed to address two relevant, though yet unresolved, issues related to the GI pathophysiology in PD: (1) the impact of nigrostriatal dopaminergic neurodegeneration on colonic tachykininergic excitatory motility and (2) the occurrence of inflammatory responses in the large bowel and abdominal cavity following the induction of nigrostriatal dopaminergic degeneration.

## Methods

### Animals

Male Sprague-Dawley rats, 200–250 g body weight, were used throughout the study. The animals were fed standard laboratory chow and tap water ad libitum and were not employed for at least 1 week after their delivery to the laboratory. They were housed, three in a cage, in temperature-controlled rooms on a 12-h light cycle at 22–24 °C and 50–60 % humidity. Their care and handling were in accordance with the provisions of the European Community Council Directive 86-609 and recognized and adopted by the Italian Government.

### Induction of nigrostriatal neurodegeneration

Animals were anesthetized with 50 mg/kg of sodium thiopental (i.p.) and placed into a stereotaxic frame (Stoelting, Wood Dale, IL, USA). Rats received 6-OHDA (dissolved in saline solution containing 0.02 % of ascorbic acid) or saline (controls) unilaterally into two sites of the right MFB, at the following coordinates (mm) relative to bregma and dural surface: (i) antero-posterior (AP) = −4.0, medio-lateral (ML) = −0.8, dorso-ventral (DV) = −8.0 tooth bar at +3.4 (9 μg/3 μL) and (ii) AP = −4.4, ML = −1.2, DV = −7.8 tooth bar at −2.4 (7.5 μg/3 μL)[10]. The injection rate was 1 μL/min using a Hamilton 10 μL syringe mounting a 26-gauge needle. After injection, the needle was left in place for 5 min to avoid leaks. At the end of process, wounds were clipped and animals allowed to wake up and recover. Animals were euthanized by cervical dislocation 4 and 8 weeks following 6-OHDA injection. Brains were immediately removed, frozen on dry ice, and stored at −80 °C, while colonic specimens were dissected and processed for functional experiments and other assays as described below. Animals injected with 6-OHDA vehicle and sacrificed after 8 weeks were selected as controls, since preliminary experiments, performed with the specific aim of comparing animals injected with 6-OHDA vehicle and sacrificed at 4 and 8 weeks, did not show any significant difference in electrically evoked colonic tachykininergic contractions (data not shown).

The 4- and 8-week time points for the evaluation of colonic motility and inflammatory response were selected on the basis of previous experiences with the experimental model of central dopaminergic degeneration induced by 6-OHDA. In particular, Blandini et al. [10] observed that rats with central dopaminergic neurodegeneration developed significant colonic functional and neurochemical changes after 4 weeks from toxin injection. In addition, in an attempt of documenting further putative colonic changes occurring in the late phases of the disease, we decided to include the 8-week group, as representative of an advanced stage of intestinal motor dysfunction associated with central dopaminergic degeneration.

### Immunohistochemistry of tyrosine hydroxylase in brain sections

Serial coronal sections (40 μm), including striatum and substantia nigra pars compacta (SNc) from both sham-operated and 6-OHDA animals, were cut on a freezing sliding microtome and mounted on polylysine-coated slides. Immunohistochemical staining for tyrosine hydroxylase (TH) was carried out to evaluate dopaminergic terminal damage in the striatum and loss of cell bodies in the SNc, as previously described [10]. Briefly, sections containing the striatum and SNc were post fixed in cold, 4 % neutral-buffered formaldehyde (NBF; Carlo Erba, Milan, Italy), rinsed in Tris-buffered saline (TBS), treated with 3 % H_2_O_2_, and incubated in TBS containing 10 % normal goat serum together with 0.6 % Triton X-100 for 30 min at room temperature. Sections were incubated overnight at 4 °C with a mouse anti-TH antibody (1:2000; Chemicon International, Temecula, CA, USA), then rinsed in TBS and incubated for 60 min at room temperature, with a goat biotinylated anti-mouse IgG antibody (1:1000; Vector Laboratories, Burlingame, CA, USA). Finally, sections were processed with the avidin-biotin technique using a commercial kit (Vectastain ABC Elite kit, Vector laboratories), and reaction products were developed using nickel-intensified 3,3′-diaminobenzidine tetrahydrochloride (DAB Substrate Kit for Peroxidase, Vector Laboratories). After rinsing with TBS, sections were dehydrated in ascending alcohol concentrations, cleared in xylene (Carlo Erba, Milan, Italy), and coverslipped.

### Evaluation of nigrostriatal neurodegeneration

The striatal dopaminergic terminal damage resulting from 6-OHDA infusion into the MFB was detected by the absence of TH staining within the striatum and expressed as the percentage of striatal volume deprived of TH immunoreactivity, as compared with the overall striatal volume. The striatal expression of TH was also evaluated in the brain of sham-operated animals. The total number of dopaminergic cells in SNc of both hemispheres was counted using stereological analysis. Unbiased stereological estimation was made using the optical fractionator method [18] by the STEREO INVESTIGATOR software on a Neurolucida computer-controlled microscopy system (Microbrightfield Inc., VT, USA). The boundaries of SNc at all levels in the rostro-caudal axis were defined, with reference to a coronal atlas of the rat brain [19]. TH-positive cells in the SNc were counted in every fourth section, on comparable sections for all experimental groups throughout the whole nucleus. Counting frames (75 × 75 μm) were placed at the intersections of a grid (frame size 208.65 × 161.6 μm) that had been randomly placed over the section. Cells were marked if they were TH-positive and were in focus within the counting area. Results represent the percentage of the number of TH-positive neurons in the injected SNc with respect to the intact side (neuron survival). We set 98 % as cut-off level for the striatal lesion and 95 % for the lesion in the SNc.

### Recording of contractile activity

The contractile activity of colonic muscle preparations was recorded as previously described by Fornai et al. [9]. Following sacrifice, the abdomen was immediately opened and the colon was removed by an incision immediately above the anal end and placed into Krebs solution. The distal colon was anatomically distinguished from proximal colon by the absence of oblique striae on the mucosal surface [20]. Longitudinal muscle strips obtained from distal colon, approximately 3-mm wide and 20-mm long, were set up in organ baths containing Krebs solution, at 37 °C, bubbled with 95 % O_2_ + 5 % CO_2_. The strips were oriented along the longitudinal axis and were connected to isometric force transducers (2Biological Instruments, Besozzo, VA, Italy); a tension of 1.0 g was then slowly applied to the preparations. The mechanical activity was recorded by BIOPAC MP150 (2Biological Instruments, Besozzo, VA, Italy). The Krebs solution had the following composition (mM): NaCl 113, KCl 4.7, CaCl_2_ 2.5, KH_2_PO_4_ 1.2, MgSO_4_ 1.2, NaHCO_3_ 25, glucose 11.5 (pH 7.4 ± 0.1). Each preparation was allowed to equilibrate for at least 30 min, with intervening washings at 10-min intervals. A pair of coaxial platinum electrodes was positioned at a distance of 10 mm from the longitudinal axis of each preparation to deliver transmural electrical stimulation by a BM-ST6 stimulator (Biomedica Mangoni, Pisa, Italy). Electrical stimuli were applied as follows: 10-s single trains (ES), consisting of square wave pulses (0.5 ms, 30 mA). At end of the equilibration period, each preparation was repeatedly challenged with electrical stimuli (10 Hz), and experiments started when reproducible responses were obtained (usually after two or three stimulations). Frequency-response curves, at frequencies ranging from 1 to 20 Hz, were constructed under the different in vitro experimental conditions reported below.

In the first series of experiments, contractions were assessed in colonic preparations maintained in Krebs solution containing N^ω-^nitro-L-arginine methylester (L-NAME) (100 μM), guanethidine (10 μM), atropine (1 μM), GR159897 (NK_2_ receptor antagonists, 1 μM), and SB218795 (NK_3_ receptor antagonists, 1 μM), in order to examine the patterns of colonic excitatory motor responses mediated by tachykininergic NK_1_ receptor pathways.

In the second series, the contractile responses were induced by direct pharmacological activation of NK_1_ tachykininergic receptors located on smooth muscle cells. For this purpose, colonic preparations were maintained in Krebs solution containing tetrodotoxin (TTX, 1 μM) and stimulated with exogenous substance P (SP, 0.001–10 μM).

### Immunohistochemistry of GFAP, SP, and NK_1_

Sections from formalin-fixed full-thickness colonic samples were processed for immunoperoxidase staining, as previously described [21]. Briefly, sections were incubated overnight at 4 °C with primary antibodies against glial fibrillary acidic protein (GFAP; code n. Z0334, DakoCytomation, Glostrup, Denmark), SP (code n. Sc-21715, Santa Cruz Biotech, California, USA), or NK_1_ (code n. orb 11133 Biorbyt Limited, Cambridge, UK) and then exposed to appropriate biotinylated immunoglobulins, peroxidase-labeled streptavidin complex, and 3,3′-diaminobenzidine tetrahydrochloride (DakoCytomation, Glostrup, Denmark). For quantitative evaluation, the immune stained sections were examined by a Leica DMRB light microscope, and representative photomicrographs were taken by a DFC480 digital camera (Leica Microsystems, Cambridge, UK). Antigen expression was evaluated as percentage of positive pixels (PPP) calculated on the total area examined of myenteric ganglia for GFAP and SP and longitudinal muscle for NK_1_ receptors. These values were used to calculate mean values for each experimental groups.

### Histological evaluation of eosinophils and mast cells

Sections from formalin-fixed full-thickness colonic samples were processed for routine (hematoxylin and eosin) and histochemical staining (0.1 % toluidine blue in 30 % ethanol for 15 min) in order to detect mast cells and eosinophils, which appeared as deep violet cells. The density of eosinophils and mast cells was evaluated in the *tunica mucosa/submucosa*. Cells were counted in three different sections from each rat, and at least 20 randomly selected microscopic fields were examined in each section (objective, ×40). Values obtained from all the examined fields for each rat were averaged and expressed as cell number per square millimeter of *tunica mucosa/submucosa* areas, which were estimated by the Image Analysis System “L.A.S. software v.4.” These values were then used to calculate mean values for each experimental group.

### Evaluation of tissue MDA levels

Malondialdehyde (MDA) concentration in specimens of colonic neuromuscular tissues was evaluated to obtain a quantitative estimation of membrane lipid peroxidation, and the assay was performed as previously described [22]. Colonic tissues were weighed, minced by forceps, homogenized in 2 ml of cold buffer (20 mMRipa buffer, pH 7.4) by a polytron homogenizer (QIAGEN), and spun by centrifugation at 1600*g* for 10 min at 4 °C. Colonic MDA concentrations were determined with a kit for colorimetric assay (Calbiochem, San Diego, CA), and the results were expressed as nmol of MDA per milligram of colonic tissue.

### Evaluation of tissue TNF and IL-1β levels

TNF and IL-1β levels in colonic neuromuscular tissues were measured by enzyme-linked immunosorbent assay kits (Abcam), as previously described [22]. For this purpose, colonic tissue samples, stored previously at −80 °C, were weighed, thawed, and homogenized in 0.4 ml of PBS, pH 7.2/20 mg of tissue at 4 °C and centrifuged at 10,000*g* for 5 min. Aliquots (100 μL) of supernatants were then used for assay. Tissue TNF and IL-1β levels were expressed as picograms per gram of tissue and picograms per milligram of proteins, respectively.

### Isolation of peritoneal macrophages

Rat peritoneal macrophages were harvested as previously described [23]. Macrophages were collected from 6-OHDA and control rats, following sacrifice, 4 and 8 weeks after 6-OHDA or saline injection. Cells were cultured as a monolayer in RPMI 1640 medium (Cellgro, Mediatech Inc., Herndon, VA), supplemented with 10 % fetal bovine serum (Gemini Bio-Products, Calabasas, CA), 2 mM L-glutamine, 100 IU/ml penicillin, and 100 μg/ml streptomycin (Irvine Scientific, Santa Ana, CA). Monolayers were washed 4 h after plating to remove non-adherent cells. Cultures were shown to be >98 % pure macrophages, as assessed by staining of non-specific esterase and macrophage-specific F4/80 mAb (data not shown).

### RNA extraction and RT-qPCR

Total RNA was isolated by means of TRIzol reagent (Invitrogen, Life Technologies, Thermo Fisher Scientific Inc., CA, USA). The concentration of isolated RNA was determined with a spectrophotometer, and 2 μg of RNA were used for the reverse transcription (RT) procedure (High Capacity cDNA Reverse Transcription Kit, Applied Biosystems, Thermo Fisher Scientific Inc., CA, USA). RT was performed in a mixture of 2 μL RT buffer, 0.8 μL dNTP, 2 μL random primer, 1 μL reverse transcriptase, and nuclease-free water (Amresco LLC, Solon, USA) up to 10 μL. PCR cycles (Veriti 96-Well Thermal Cycler, Applied Biosystems, Thermo Fisher Scientific Inc., CA, USA) were set as follows: 25 °C for 10 min, 37 °C for 120 min, and 85 °C for 5 min. Tenfold dilutions of complementary DNA (cDNA) specimens were used for the real-time quantitative PCR analysis (RT-qPCR) procedure. qPCR reactions were performed with the following primers: inducible nitric oxide synthase (iNOS) 5′-CCTTGTTCAGCTACGCCTTC-3′ (sense) and 5′-CCAGGCCAAATACCGCATAC-3′ (anti-sense); arginase-1 5′-GGACATCGTGTACATCGGCT-3′ (sense) and 5′-GGGCCTTTTCTTCCTTCCCA-3′ (anti-sense); 18S 5′-GGGAGCCTGAGAAACGGC-3′ (sense) and 5′-GGGTCGGGAGTGGGTAATTTT-3′ (anti-sense). SYBR Green (Applied Biosystems, Thermo Fisher Scientific Inc., CA, USA) qPCR reactions were run in Light Cycler 480 II Real-Time PCR Instrument (Roche, Basel, Switzerland). Expression values were normalized to the housekeeping gene 18S expression.

### Drugs and reagents

Atropine sulfate, guanethidine monosulphate, N^ω-^nitro-L-argininemethylester, 6-hydroxy dopamine, and ascorbic acid were purchased from Sigma Chemicals Co. (St. Louis, MO, USA). Tetrodotoxin, Substance P, N-acetyl-L-tryptophan 3,5-bis (trifluoromethyl) benzyl ester, GR159897, and SB218795 were purchased from Tocris (Bristol, UK). Mouse anti-TH antibody (1:2000) was purchased from Chemicon International (Temecula, CA, USA). Biotinylated anti-mouse IgG antibody (1:1000) and nickel-intensified 3,3′-diaminobenzidine tetrahydrochloride (DAB Substrate Kit for peroxidase) were purchased from Vector Laboratories (Burlingame, CA, USA). Neutral-buffered formaldehyde (NBF) and xylene were purchased from Carlo Erba (Milan, Italy).

### Statistical analysis

The results are presented as mean ± S.E.M. unless otherwise stated. The significance of differences was evaluated by Student *t* test for unpaired data or one-way analysis of variance (ANOVA) followed by post hoc analysis with Student-Newman-Keuls or Bonferroni tests. *P* values <0.05 were considered significantly different. All statistical procedures were performed by commercial software (GraphPad Prism, version 3.0 from GraphPad Software Inc., San Diego, CA, USA).

## Results

### Immunohistochemical analysis of TH in brain

As previously observed [9], the unilateral injection of 6-OHDA into the MFB caused a virtually complete loss of dopaminergic striatal terminals (98 %) and dopaminergic nigral neurons (95 %) of the right (injected) hemisphere, both at weeks 4 and 8. Animals bearing lesions of less than 98 % in the striatum and 95 % in the SNc were excluded from the study. Sham-operated rats did not display differences in TH immunoreactivity between hemispheres, both at weeks 4 and 8 (data not shown).

### In vitro colonic contractile activity

**Fig. 1:**
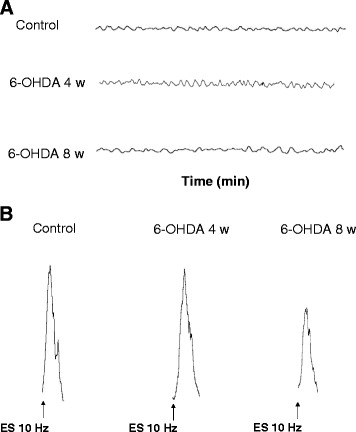
Longitudinal smooth muscle preparations of distal colon isolated from control animals or 6-OHDA rats at weeks 4 and 8 and maintained in standard Krebs solution. **a** Representative tracings displaying colonic spontaneous motor activity under resting conditions. **b** Representative tracings displaying colonic contractile responses elicited by electrical stimulation (ES: 0.5 ms, 30 mA, 10 s, 10 Hz)

During equilibration, most distal colonic preparations displayed a rapid spontaneous motor activity, which was low in amplitude and generally stable throughout the experiment (Fig. 1a).

No significant differences in spontaneous motor activity were observed when comparing controls (mean amplitude: 2.3 g/g tissue) to animals injected with 6-OHDA, both at weeks 4 and 8 (mean amplitude 2.7 and 2.5 g/g tissue, respectively). Electrically evoked responses consisted of phasic contractions followed, in some cases, by after-contractions of variable amplitude (Fig. 1b). In colonic preparations from control animals and maintained in standard Krebs solution, the contractions evoked by electrical stimulation accounted for 45.2 ± 3.5 g/g tissue (at 10 Hz). In tissues from 6-OHDA-treated rats after 4 weeks, electrically evoked contractions did not differ significantly (44.2 ± 1.5 g/g tissue, 10Hz), while they were significantly reduced in animals after 8 weeks from 6-OHDA injection (32.9 ± 2.2 g/g tissue, 10 Hz, *P* < 0.05).

**Fig. 2:**
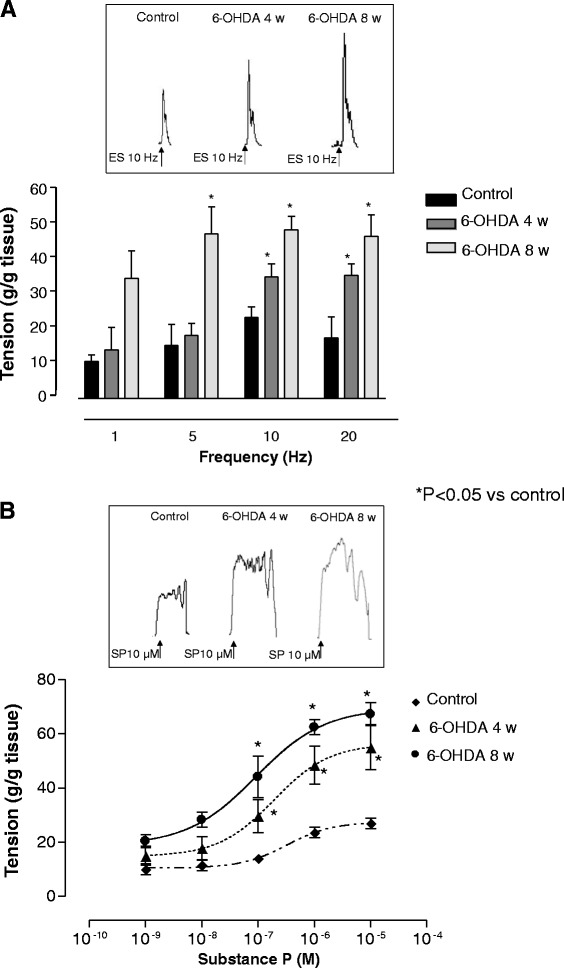
Longitudinal smooth muscle preparations of distal colon isolated from control animals or 6-OHDA rats at weeks 4 and 8. **a** Tissues were maintained in Krebs solution, containing L-NAME (100 μM), guanethidine (10 μM), atropine (1 μM), GR159897 (NK2 receptor antagonists, 1 μM), and SB218795 (NK3 receptor antagonists, 1 μM), and contractile responses were elicited by electrical stimulation (ES: 0.5 ms, 30 mA, 10 s, 1–20 Hz). Each column represents the mean ± S.E.M value obtained from eight experiments. *P < 0.05, significant difference vs control. Tracings in the inset on the top of each panel display contractile responses to ES recorded at a frequency of 10 Hz. **b** Tissues were maintained in standard Krebs solution containing tetrodotoxin (1 μM), and contractions were evoked by increasing concentration of exogenous substance P (SP, 0.001–10 μM). Tracings in the inset on the top of each panel display contractile responses to exogenous SP at a concentration of 10 μM. Each point represents the mean ± S.E.M value (*n* = 8). *P < 0.05, significant difference vs control

In longitudinal muscle preparations from distal colon, maintained in Krebs solution containing L-NAME (100 μM), guanethidine (10 μM), atropine (1 μM), GR159897 (NK_2_ receptor antagonists, 1 μM), and SB218795 (NK_3_ receptor antagonists, 1 μM), the electrically evoked NK_1_-mediated tachykininergic contractions were enhanced after 4 weeks (significant difference at 10 and 20 Hz) and 8 weeks (significant difference at 5, 10 and 20 Hz), as compared with controls (Fig. 2a). L-732.138 (NK_1_ receptor antagonist, 10 μM) almost completely abolished these phasic contractions (90 %).

The application of exogenous substance P (SP, 0.001-10 μM) to longitudinal smooth muscle preparations from distal colon in the presence of tretrodotoxin (1 μM) evoked concentration-dependent concentrations, which were enhanced 4 and 8 weeks after 6-OHDA injection, as compared with controls (Fig. 2b).

### Quantitative immunostaining of GFAP, SP, and NK_1_ in the colonic neuromuscular layer

**Fig. 3:**
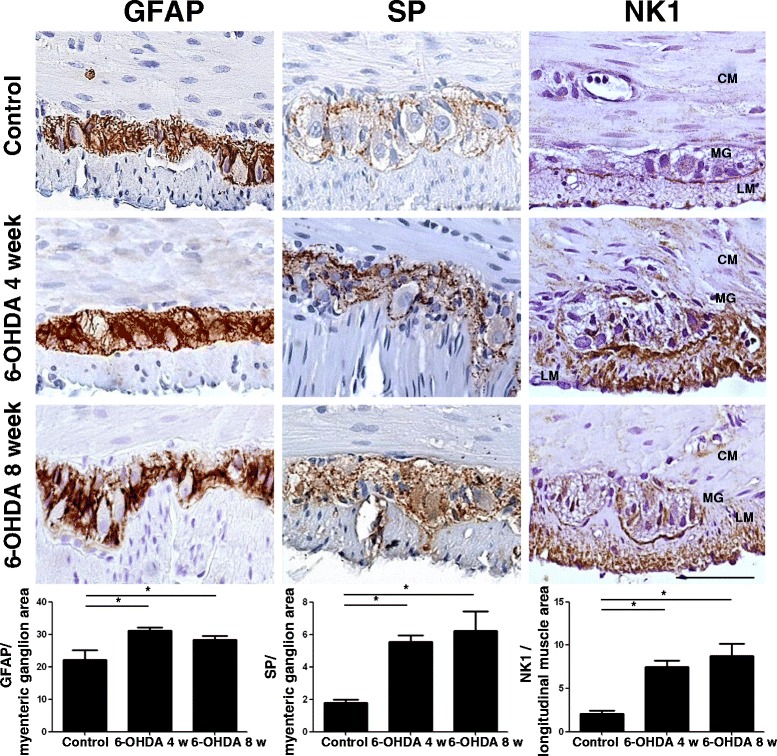
Representative pictures of GFAP, SP, and NK_1_ immunostaining of cross-sections from full-thickness rat colonic specimens from controls or 6-OHDA animals at weeks 4 and 8. Scale bar = 50 μm. Column graphs display the quantitative assessment of GFAP and SP immunostaining in myenteric ganglion area and NK_1_ receptors in longitudinal muscle area. Each column represents the mean value of PPP ± S.E.M. value (*n* = 8). *P < 0.05. *CM* circular muscle, *LM* longitudinal muscle

Glial cells of colonic myenteric ganglia displayed GFAP immunopositivity that was significantly increased in rats with 6-OHDA-induced nigrostriatal denervation at both weeks 4 (+41 % vs control) and 8 (+28 % vs control). Likewise, a significant increase in SP immunostaining was found in myenteric ganglia of 6-OHDA animals (+209 % vs control after 4 weeks and +247 % vs control after 8 weeks), along with an enhancement of NK_1_ receptor expression in the longitudinal muscle layer, both at weeks 4 (+201 % vs control) and 8 (+328 % vs control) (Fig. 3).

### Assessment of MDA, TNF, and IL-1β levels in colonic neuromuscular tissues

**Fig. 4:**
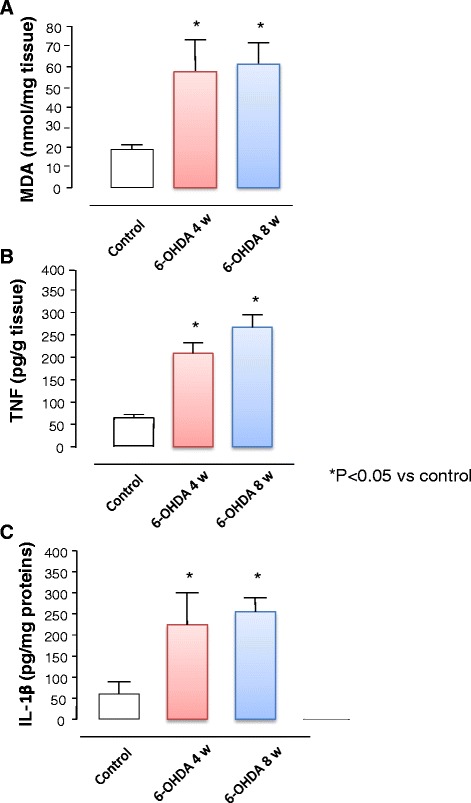
MDA (**a**), TNF (**b**), and IL-1β (**c**) levels in colonic tissues from control animals and rats after 4 and 8 weeks from nigrostriatal degeneration with 6-OHDA. Each column represents the mean ± S.E.M value (*n* = 8). *P < 0.05, significant difference vs control

MDA levels in colonic specimens from control rats accounted for 18.8 ± 2.4 nmol/mg tissue (Fig. 4a). 6-OHDA-induced nigrostriatal degeneration was associated with a significant increase in the oxidative stress of colonic tissues at both weeks 4 (57.6 ± 15.8 nmol/mg tissue; +206.3 % vs control) and 8 (61.4 ± 10.7 nmol/mg tissue; +226.6 % vs control) (Fig. 4a).

TNF levels in colonic tissues from control animals accounted for 64.7 ± 7.2 pg/g tissue (Fig. 4b). In colonic tissues from rats with 6-OHDA-induced nigrostriatal degeneration, TNF levels were increased at both weeks 4 (208.6 ± 23.7 pg/g tissue; +222.4 %) and 8 (267.4 ± 27.5 pg/g tissue; +313.2 %), as compared with controls (Fig. 4b).

IL-1β levels in colonic tissues from control animals accounted for 60.02 ± 16.2 pg/mg proteins (Fig. 4c). In colonic tissues from rats with 6-OHDA-induced nigrostriatal degeneration, IL-1β levels were increased at both weeks 4 (224.5 ± 56.2 pg/mg proteins; +274.1 %) and 8 (255.5 ± 28.1 pg/mg proteins; +325.7 %), as compared with controls (Fig. 4c).

### Tissue distribution and density of eosinophils and mast cells in the colonic wall

**Fig. 5:**
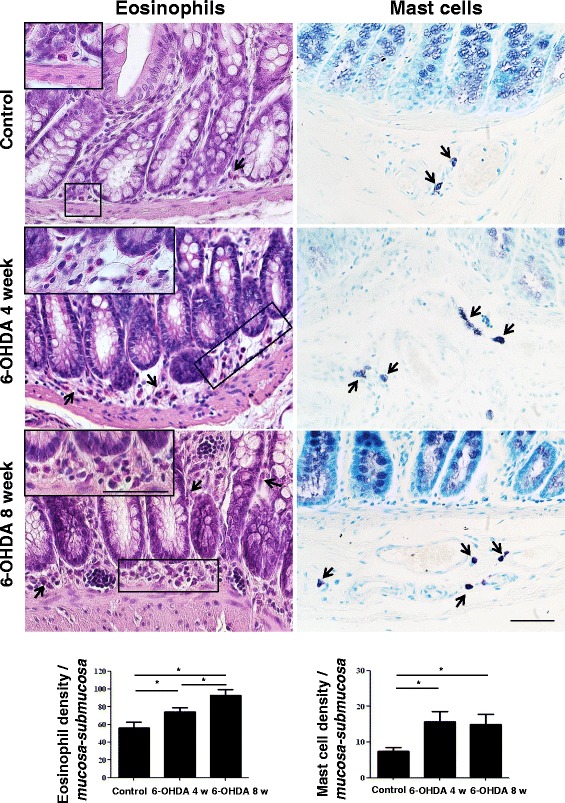
Representative pictures of hematoxylin/eosin- and toluidine blue-stained colonic sections obtained from controls or animals treated with 6-OHDA at weeks 4 and 8. The column graphs display mean values of eosinophil or mast cell density per square millimeter of *tunica mucosa/submucosa* areas (cells/mm^2^) ± S.E.M. value (*n* = 8). *P < 0.05. Scale bars = 50 μm

Eosinophils, which were frequently found within the *tunica mucosa* and *submucosa* of normal colon, were significantly increased in 6-OHDA rats, both at weeks 4 (+32 % vs control) and 8 (+66 % vs control, and +25 % vs 6-OHDA rats at 4 week), which displayed also sporadic eosinophils in the *tunica muscularis* and along the myenteric ridge (Fig. 5). Mast cells, which were occasionally detected in controls within the perivascular connective tissue of *tunica mucosa*, *submucosa*, and *serosa*, were significantly increased in density in 6-OHDA-treated rats at both time points (+111 and +102 % vs control, respectively) (Fig. 5).

### Pro-inflammatory polarization of peritoneal macrophages

**Fig. 6:**
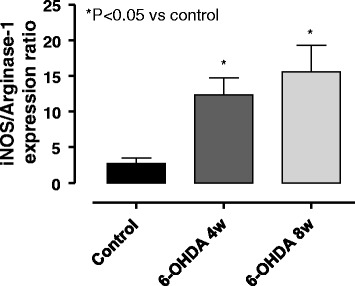
iNOS/arginase-1 expression ratios in peritoneal macrophages from control rats and 6-OHDA animals at weeks 4 and 8. Each column represents the mean ± S.E.M. value (*n* = 8). *P < 0.05, significant difference vs control

Peritoneal macrophages from normal rats have been previously shown to express higher levels of iNOS mRNA than arginase-1 mRNA [24]. Accordingly, peritoneal macrophages isolated from control rats displayed aiNOS/arginase-1 expression ratio accounting for 2.7 ± 0.3 (Fig. 6). In peritoneal macrophages obtained from 6-OHDA rats at weeks 4 and 8, the iNOS/arginase-1 expression ratio (reminiscent of macrophage M1/M2 polarization) was significantly increased, as compared control rats (12.2 ± 2.4 and 15.8 ± 3.7, respectively) (Fig. 6).

## Discussion

To extend current knowledge about changes in the enteric excitatory pathways regulating colonic motility under PD, we examined the impact of nigrostriatal dopaminergic degeneration on colonic tachykininergic contractile activity. The present findings support the view that the induction of nigrostriatal neurodegeneration, a condition which reflects one of the major pathological hallmarks of PD, is followed by significant alterations of distal colonic excitatory tachykininergic neuromotility. In particular, we observed an increase in the electrically evoked tachykininergic contractions of longitudinal muscle preparations from 6-OHDA rats, recorded upon incubation of colonic tissues with atropine, guanethidine, L-NAME, and neurokinin NK_2_ and NK_3_ receptor antagonists, as well as an enhancement of myogenic motor responses evoked by exogenous application of SP in the presence of tetrodotoxin to abate neurogenic responses. Of note, electrically evoked contractions, recorded from preparations maintained in standard Krebs solution, were reduced in 6-OHDA rats as compared with controls (see Fornai et al. [9]), despite an increase in tachykininergic neuromotility, as observed in the present experiments. Analogously, our previous in vivo observation of a reduced colonic transit in 6-OHDA rats is also apparently at odds with the present in vitro results showing an enhanced tachykininergic neuromuscular activity [9]. However, in our previous study we observed a significant impairment of in vitro cholinergic neuromotor activity. In addition, the overall colonic propulsive motility is known to result from an integrated combination of circular and longitudinal smooth muscle contractions regulated by both excitatory and inhibitory neurotransmitter pathways [25]. Therefore, it is conceivable that the overall contractions recorded from colonic longitudinal muscle preparations maintained in standard Krebs solution, as well as the patterns of in vivo colonic transit, do not necessarily reflect the patterns of contractile responses elicited by specific stimulation of the excitatory tachykininergic pathway.

In the present study, efforts were then dedicated to investigate the mechanisms underlying the enhancement of colonic tachykininergic motility in 6-OHDA rats. As a first step, we examined whether these motor changes could depend on variations of the density of myenteric tachykininergic neurons. This goal was pursued by immunohistochemical assays, where myenteric ganglia were labeled with an anti-SP antibody. In these experiments, colonic tissues from 6-OHDA rats displayed a significant increase in immunopositivity for SP in myenteric ganglia. Since the overall density of neurons was previously shown to not vary in 6-OHDA rat model [9], our findings suggest that nigrostriatal denervation is associated with an increased SP expression in myenteric tachykininergic neurons, which could contribute to the enhancement of colonic tachykininergic contractile responses, as shown in our functional experiments. Besides the increase in electrically evoked tachykininergic contractions of distal colon in 6-OHDA rats, we detected also an enhancement of colonic myogenic contractions elicited by a direct activation of tachykininergic NK_1_ receptors with exogenous SP. To better interpret these results, we went on to examine the expression of NK_1_ receptors in colonic specimens by immunohistochemistry, observing an up-regulation of this receptor subtype in the colon of 6-OHDA rats. To the best of our knowledge, these experiments provide the first demonstration of an altered colonic tachykininergic neuromuscular pattern in an experimental model of nigrostriatal dopaminergic neurodegeneration. Interestingly, a similar picture has been previously observed in brain tissues from rats with 6-OHDA-induced central dopaminergic degeneration, where an increased SP expression was detected [26]. Overall, based on the present findings, it is conceivable that an enhancement of tachykininergic enteric neurotransmission, supported by an increased expression of both SP and NK_1_ receptors, could contribute to the colonic motor abnormalities occurring in 6-OHDA rats.

A number of previous studies have reported that changes in enteric tachykininergic pathways are involved in the pathophysiology of motor GI disorders associated with inflammatory conditions (i.e., inflammatory bowel diseases, diverticulitis, and irritable bowel syndrome) [13, 14]. In particular, an increased SP release has been observed from neurons of myenteric and submucosal plexuses, as well as from immune cells of the intestinal *lamina propria* (such as monocytes, macrophages, eosinophils, and lymphocytes), isolated from patients with ulcerative colitis or Crohn’s disease, leading to the hypothesis that the enteric tachykininergic system is involved in the pathogenesis of bowel motor dysfunctions associated with inflammation [27]. For these reasons, and considering also previous observations made in bowel tissue specimens from PD patients [17], in the second part of the present study, we focused our attention on investigating the presence of inflammatory processes in rat colonic tissues following central dopaminergic neurodegeneration. Interestingly, our results highlighted clear signs of colonic inflammation in tissues isolated from 6-OHDA rats, characterized by increased levels of MDA, TNF, and IL-1β, thus suggesting the presence of gut inflammation and oxidative stress in the colonic wall. Taken together, these findings provide the first demonstration that after nigrostriatal degeneration the distal colon undergoes a condition of inflammatory activation. The presence of bowel inflammation in 6-OHDA rats was further corroborated by an increase in eosinophil and mast cell density within the colonic *tunica mucosa* and *submucosa*, as well as by the evidence of glia activation in myenteric ganglia. Of interest, our results are in line with previous clinical findings displaying an increase in pro-inflammatory cytokine concentrations, including TNF and IL-1β, and markers of glial cell activation (i.e., increase in ganglionic GFAP levels) in colonic biopsies from PD patients [17].

Previous studies have shown that the dorsal motor nucleus of the vagus (DMV)-vagus nerve axis, one of the earliest CNS sites affected by PD pathology, is significantly involved in the pathophysiology of visceral inflammation [28, 29]. Vagal anti-inflammatory signaling within the GI tract is mediated by activation of nicotinic receptors [30, 31]. A decay of this signaling—as that occurring in PD—might favor conditions of visceral inflammation that may play a substantial role in PD-related GI dysfunctions [32]. Therefore, we hypothesized that central dopaminergic neurodegeneration triggered by 6-OHDA might lead to an inflammatory abdominal condition through an impairment of the DMV-vagus nerve anti-inflammatory pathway. To substantiate this hypothesis, we examined also peritoneal macrophages, which are known to polarize towards the M1 pro-inflammatory phenotype, characterized by an increased expression of iNOS (inducible nitric oxide synthase), or the M2 “wound healing” phenotype, in which cells express high levels of arginase-1 [24, 33]. This series of experiments showed that at both 4 and 8 weeks after 6-OHDA injection, peritoneal macrophages were significantly polarized towards the M1 pro-inflammatory phenotype, characterized by a high iNOS/arginase-1 expression ratio, thus corroborating the concept that 6-OHDA animals undergo a condition of abdominal visceral inflammation following dopaminergic intranigral neurodegeneration.

Based on the present findings, it is conceivable that an enhancement of tachykininergic enteric neurotransmission, supported by an increased expression of both SP and NK_1_ receptors, as well as the presence of colonic inflammation, could contribute to the enteric motor abnormalities occurring after nigrostriatal dopaminergic degeneration. In this respect, increasing evidence supports the occurrence of a close interplay between the SP/NK_1_ pathway and the onset of immune/inflammatory responses [27, 34]. In particular, studies on human colonic epithelial cell lines as well as on intestinal tissues from patients with inflammatory bowel diseases revealed that inflammatory cytokines, such as TNF and IL-1β, promoted an up-regulation of NK_1_ receptor expression and a marked increase in SP release. In turn, such a massive increment of SP activity, through an induction of pro-inflammatory signaling in immune cells (i.e., mitogen-activated protein kinases and nuclear factor-kB), has been shown to increase the secretion of pro-inflammatory cytokines (i.e., IL-1β, IL-6, and TNF), thus generating a positive feedback loop that could drive the chronicization of the ongoing inflammatory process [27, 34].

## Conclusions

In conclusion, our findings indicate that central dopaminergic nigrostriatal degeneration is associated with the occurrence of colonic inflammation and an enhancement of excitatory tachykininergic neurotransmission, which may contribute to bowel motor alterations. These findings can provide a basis for better understanding the mechanisms underlying GI motor abnormalities in PD, thereby paving the way to the development of suitable pharmacological treatments for the management of GI functional disorders associated with PD.

## Abbreviations

6-OHDA, 6-hydroxydopamine; DMV, dorsal motor nucleus of the vagus; GFAP, glial fibrillary acidic protein; GI, gastrointestinal; IL-1β, interleukin1-beta; MDA, malondialdehyde; MFB, medial forebrain bundle; PD, Parkinson’s disease; SNc, substantia nigra pars compacta; SP, substance P; TH, tyrosine hydroxylase; TNF, tumor necrosis factor; TTX, tetrodotoxin
